# Function of CTLGA9 Amino Acid Residue Leucine-6 in Modulating Cry Toxicity

**DOI:** 10.3389/fimmu.2022.906259

**Published:** 2022-07-05

**Authors:** Intikhab Alam, Khadija Batool, Aisha Lawan Idris, Weilong Tan, Xiong Guan, Lingling Zhang

**Affiliations:** ^1^State Key Laboratory of Ecological Pest Control for Fujian and Taiwan Crops, College of Life Sciences, Fujian Agriculture and Forestry University, Fuzhou, China; ^2^Key Lab of Biopesticides and Chemical Biology, MOE, Fujian Agriculture and Forestry University, Fuzhou, China; ^3^College of Life Sciences, South China Agricultural University, Guangzhou, China; ^4^Nanjing Bioengineering (Gene) Technology Center for Medicines, Nanjing, China

**Keywords:** *Aedes aegypti*, lectins, receptor, mutation, Cry11Aa, toxicity

## Abstract

*Aedes aegypti* is a crucial vector for many arboviral diseases that cause millions of deaths worldwide and thus is of major public health concern. Crystal (Cry) proteins, which are toxins produced by *Bacillus thuringiensis*, are structurally organized into three-domains, of which domain II is the most variable in terms of binding towards various toxin receptors. The binding of Cry11Aa to putative receptor such as aminopeptidase-N (APN) is explicitly inhibited by midgut C-type lectins (CTLs). The similarity between the domain II fold of Cry11Aa toxin and the carbohydrate recognition domain in the CTLs is a possible structural basis for the involvement of Cry domain II in the recognition of carbohydrates on toxin receptors. In this study, a site-directed point mutation was introduced into the *A. aegypti* CTLGA9 gene on the basis of molecular docking findings, leading to substitution of the Leucine-6 (Leu-6) residue in the protein with alanine. Subsequently, functional monitoring of the mutated protein was carried out. Unlike the amino acid residues of wild-type CTLGA9, none of the residues of mutant (*m*) CTLGA9 were competed with Cry11Aa for binding to the APN receptor interface. Additionally, ligand blot analysis showed that both wild-type and mutant CTLGA9 had similar abilities to bind to APN and Cry11Aa. Furthermore, in the competitive ELISA in which labeled mutant CTLGA9 (10 nM) was mixed with increasing concentrations of unlabeled Cry11Aa (0–500 nM), the mutant showed no competition with Cry11Aa for binding to APN., By contrast, in the positive control sample of labeled wild type CTLGA9 mixed with same concentrations of Cry11Aa competition between the two ligands for binding to the APN was evident. These results suggest that Leucine-6 may be the key site involved in the competitive receptor binding between CTLGA9 and Cry11Aa. Moreover, according to the bioassay results, mutant CTLGA9 could in fact enhance the toxicity of Cry11Aa. Our novel findings provide further insights into the mechanism of Cry toxicity as well as a theoretical basis for enhancing the mosquitocidal activity of these toxin through molecular modification strategies.

## Introduction

The mosquito *Aedes aegypti* is a crucial vector for many arboviral diseases that cause millions of deaths worldwide and thus of major public health concern (1, 2). Mosquito control is thus critical to halting the spread of these diseases (3), there is as yet no effective strategy in this regard. Traditionally, mosquito control is implemented through the use of chemical pesticides, but such compounds may lead to environmental pollution and the emergence of resistant insect species (4, 5). Recently, microbiological pesticides have been widely applied and promoted being environmentally safe and highly specific, exerting little influence on non-target organisms while resulting in lower resistance in the target species (3, 6, 7). Thus, microbial pesticides have proven to be the ideal way to control mosquitoes. *Bacillus thuringiensis is* a gram-positive, soil bacterium that, produces crystal (Cry) proteins, which are toxins that have been widely applied in agriculture to control insect pests (8). In particular, *B. thuringiensis* subspecies *israelensis* that, produces different Cry proteins capable of killing mosquitoes, including Cry4Aa, Cry4Ba, Cry10Aa, Cry11Aa, and Cyt1Aa (9, 10).

Structurally, insecticidal Cry proteins are made up of three conserved domains, each of which holds a specific function (11). Domain I, which is involved in pore formation, comprises a bundle of 7–8 alpha helices. Domain II, which carries out receptor binding activity, composed of beta-prism folds with exposed loop regions. Domain III, made up of beta-sandwich folds, is involved in receptor binding, insect specificity, and ion channel formation (12–15). The sequential binding mechanism of the Cry protein is activated when mosquito larvae are exposed to the toxin. After their solubilization and activation by proteases in the insect midgut, the Cry protein bind to specific receptors such as alkaline phosphatase (ALP), aminopeptidase-N (APN), and cadherin (CAD) which are located on brush border membrane vesicles in the insect midgut. This toxin-receptor interaction causes pore formation in the epithelial cells resulting in osmotic imbalance and cell death (11, 16, 17). Cry toxins and lectins exhibit structural similarity, in that they both have a β-prism fold structure. Therefore, lectins could interfere with the binding of Cry toxins to their receptors by binding competitively to the glycosylated receptor (18, 19).

Lectins a class of carbohydrate binding proteins, are ubiquitously expressed in plants, animals, bacteria, and viruses (20). They play a major role in the self and non-self-recognition processes of the humoral immune system. Interestingly, lectin proteins can recognize and bind to specific carbohydrate structures on the surface of the cells causing cell agglutination. s. Because some insect lectins can recognize polysaccharide chains on the surface of pathogens, they are assumed to be involved in self-defense.

Molecular simulation and molecular docking analyses can provide strong support for the study of protein-protein interactions (21). In this study, a site-directed point mutation was introduced into the *A. aegypti* CTLGA9 gene on the basis of previous protein-protein interaction studies and the prediction of hot spot residues in the ligand-receptor complex. Subsequently, the role of the predicted residue in the interaction between Cry11Aa and the receptor APN was verified using the ligand blot assay and enzyme-linked immunosorbent assay (ELISA). The findings of this study will lay a foundation for further research on the interaction between the toxin Cry11Aa and other receptors, as well as on the enhancement of the insecticidal activity of Cry toxin through their molecular modification.

## Materials and Methods

### Materials

A Haikou strain of *A. aegypti*, obtained from the Fujian International Travel Health Care Center, was reared in the laboratory under controlled conditions of 28°C, 75-80% relative humidity, and 14:10 h light: dark photoperiod. *B. thuringiensis* Cry11Aa and APN receptor strains were provided by Dr. Sarjeet Gill, University of California Riverside, CA, U.S.A. *Escherichia coli* strain DH5α and *E. coli* BL21 (DE3) competent cells, and plasmid pMD18-T were purchased from TaKaRa (Dalian, China). The expression vector pET32α used in this study was among our laboratory stock. EZ-Link-NHS-Biotin was purchased from Thermo Fisher Scientific (Waltham, MA, USA),. Streptavidin horse-radish peroxidase (HRP) conjugated antibody was purchased from Beyotime Biotech (Shanghai, China), biotin specific antibody streptavidin/AP from BIOSS Antibodies (Beijing, China), rabbit polyclonal antibodies and anti-6 × His antibody was bought from BBI Life Science (Shanghai, China). ProteinIso^®^ Ni-NTA resins were purchased from TransGen Biotech (Beijing, China).

### Modeling and Protein Docking Analyses of CTLGA9, Cry11Aa and APN

The three-dimensional protein structures of CTLGA9 (PDB: 5EAL), Cry11Aa (PDB: 1DLC), and APN (PDB: 4WZ9) were obtained *via* a modeling approach using Phyre-2 software, as reported in our previously published study (19). Protein docking simulations were conducted with the Discovery Studio 2.5. software. The docking complexes were obtained using the GRAMM-X Protein-Protein Docking Web Server v.1.2.0 (http://vakser.compbio.ku.edu/resources/gramm/grammx) and analyzed through the root mean square deviation of the receptor and binding interface. Hot spot residues were predicted based on the comparisons of docking complexes. An alanine substitution mutation was introduced in the CTLGA9 protein and the binding interaction of the mutant was further confirmed through molecular docking analysis (22). The amino acid sequence of mutant CTLGA9 was modeled using the Phyre-2 server. From the top 10 model templates obtained from the program, the first one was selected according to best coverage efficiency and confidence level. The structure was assessed geometrically using different programs, such as the Ramachandran plot in Discovery Studio and the Z-score and energy plot from the ProSAweb server (https://prosa.services.came.sbg.ac.at/prosa.php). Subsequently, docking complexes of *m* CTLGA9 with the toxin and receptor were generated and analyzed using Discovery Studio software.

### Isolation, Expression, and Purification of Mutant CTLGA9

The nucleotide sequence of *A. aegypti* wild-type CTLGA9 (*wt* CTLGA9) was retrieved from the National Center for Biotechnology Information (Genbank Accession No: AAEL014385) and submitted to the SMART database (http://smart.embl-heidelberg.de/) for domain and protein analysis. The target fragment was amplified using *wt* CTLGA9 plasmid as the template and the mutant primer pair (Forward-primer: 5′CATGCCATGGAAGCATGGAGAgcGTG3′; Reverse-primer: 5′CCCAAGCTTATCCAAGATGCAACTCCTA3′). TransStart FastPfu Fly DNA polymerase (TransGen Biotech), was used for amplification of the sequences. The purified polymerase chain reaction (PCR) product was ligated into the pMD18T vector following enzyme digestion with *NcoI* and *Hind III* was carried out. Then the fragments were further ligated to the pre-digested vector pET32α. The constructed recombinant plasmids (pET32α-CTLGA9) were transferred into *E. coli* BL21 (DE3) competent cells. The correct insertion of the *m* CTLGA9 segment was confirmed through enzyme digestion and verified by sequencing. A single colony of freshly transformed *E.coli* (BL21) cells carrying the recombinant plasmids was then cultured overnight in ampicillin containing 2YT medium, with constant agitation of 200 rpm and 37°C. Thereafter, 1% of the total volume of the overnight culture was inoculated into fresh 2YT medium until the OD_600_ reached 0.6-0.8. To induce the expression of CTLGA9, 0.5 mM isopropyl β-D-1-thiogalactopyranoside was added to the cells, and the culture was incubated for 20 h at 16°C with agitation at 200 rpm. Thereafter, the cells were collected by centrifugation (8000 rpm for 10 min at 4°C), washed, and resuspended in binding buffer (0.5 M NaCl, 20 mM sodium phosphate, 10 mM imidazole, pH: 7.4), and then lysed by sonication for 30 min to disrupt the cell membranes and release the cellular contents. Then, the lysate solution was centrifuged (11000 rpm for 15 min at 4°C). Supernatants were passed through a Ni-NTA affinity column that had been pre-equilibrated with binding buffer (0.5 M NaCl, 20 mM sodium phosphate, 10 mM imidazole, pH: 7.4). Subsequently, the protein molecules bound to Ni-NTA resins, were eluted with an elution buffer (0.5 M NaCl, 20 mM sodium phosphate, and 400 mM imidazole, pH: 7.4). Fractions containing the target protein were checked using 12% sodium dodecyl sulfate–polyacrylamide gel electrophoresis (SDS-PAGE), and the protein concentrations were measured with the Bradford method (23). The receptor protein APN was also purified using His-tagged Ni-NTA chromatography.

### Immunoblotting and ELISA Based Competitive Binding Assay

Quality checking of the recombinant protein was performed using the western blot assay (24). In brief, biotinylated *m* CTLGA9 and *wt* CTLGA9 proteins were first separated using 10% SDS-PAGE and then transferred to a polyvinylidene difluoride (PVDF) membrane. Detection of the proteinswas carried out using the biotin-specific antibodies (1:3000), following which a 5-bromo-4-chloro-3-indolyl phosphate/nitro blue tetrazolium (BCIP/NBT) kit (Beyotime, Shanghai, China) was used to confirm the results. The binding activity of *m* CTLGA9 to Cry11Aa and APN receptor was analyzed with the ligand blot assay (21). In brief, 10% SDS-PAGE separated *m* CTLGA9 protein was transferred to a PVDF membrane which was then incubated with phosphate-buffered saline (PBS) containing 5% skim milk, and subsequently washed three times with PBS containing Tween-20 (PBST). Thereafter, the mutant protein-containing membrane was incubated with Cry11Aa (10 μg/mL) or APN (10 μg/mL) proteins for 2 h and washed with PBST. Subsequently, the membranes were incubated with specific anti-Cry11Aa and anti-APN polyclonal antibodies (1:3000) for 1.5 h. Next, the membranes was washed with PBST and further incubated with secondary antibodies (1:3000) for 1.5 h. After three washes with PBST and PBS, the immune complexes were detected using the BCIP/NBT color development kit. Thioredoxin (Trx) and *wt* CTLGA9 were used as controls. The competitive ELISA was performed to confirm the binding interaction between the *m* CTLGA9 proteins and APN proteins (24). To determine whether *m* CTLGA9 and Cry11Aa competed with each other for binding to APN, a competitive ELISA was conducted following previously reported methods (19, 25). In brief, the wells of a 96-well plate were coated with APN proteins (4 μg/well), and the plate was stored at 4°C overnight. After blocking with 5% skim milk mixed with PBS buffer, 0-500 nM of unlabeled Cry11Aa proteins mixed with 10 nM labeled *m* CTLGA9 were added to the respective wells for incubation at room temperature for 1 h. Additionally, 10 nM biotin labeled Cry11Aa was mixed with increasing concentrations of unlabeled *m* CTLGA9 (0–500 nM) was added to each well of a pre-coated ELISA plate. The labeled CTLGA9 and Cry11Aa proteins were detected using the streptavidin HRP-conjugated antibody (1:3000). Then, 100 μL of the chromogenic agent 3,3′5,5′-tetramethylbenzidine (TMB) (Beyotime, Shanghai, China) was added to each well, and the plate was incubated at 37°C for 15 min. The reaction was terminated by the addition of 2M H_2_SO_4_, and absorbance of the solution in each well was recorded at 450 nm. Each treatment was replicated three times. Trx and *wt* CTLGA9 proteins mixed with Cry11Aa were used as controls. GraphPad Prism software was used to analyze the data.

### Larvae Bioassay

The *A. aegypti* larvae bioassay was performed according to the methods described in previously published report (26). In short, third instar larvae of *A. aegypti* were fed Cry11Aa protein (0.85 μg/mL) or a mixture of Cry11Aa and *m* CTLGA9 protein (0.15, 1.5, and 15 μg/mL). Each treatment was replicated three times. The survival rate of the mosquito larvae was recorded after 12 and 24 h. The Trx protein and *wt* CTLGA9 protein (0.15, 1.5, and 15 μg/mL) mixed with Cry11Aa were used as controls. Data were analyzed using the GraphPad Prism software, and each bar represents the mean ± SD of three replicates.

### Growth Curve of Mutant CTLGA9

The growth rates of recombinant ***E. coli*** strains (those expressing *m* CTLGA9, and *wt* CTLGA9) were measured and compared to with that of the Trx-expressing control strain. All strains were grown in Luria-Bertani (LB) medium containing ampicillin (100µg/mL) with constant agitation at 200 rpm, 37°C until the OD_600_ of the culture reached 0.6-0.8. Then, the bacterial culture was transferred into 100 mL of fresh LB medium. Samples were loaded into the sample tray of a fully automatic growth curve analyzer (Bioscreen C, Finland), and the growth rate was measured for 0-72 h at 37°C.

## Results

### Protein Docking Analysis of CTLGA9 and Cry11Aa With APN Receptor

**Figure 1:**
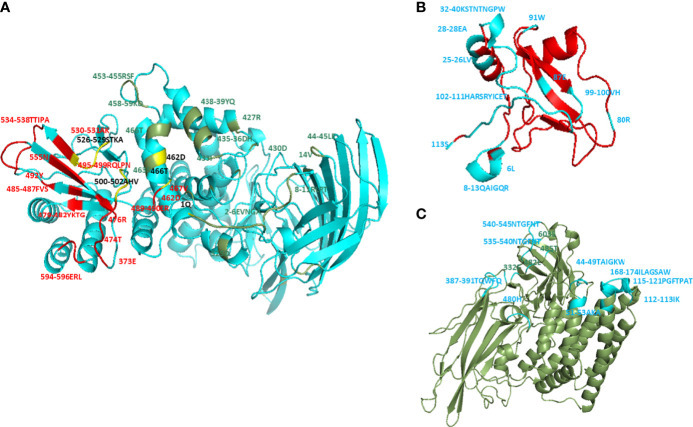
Molecular docking of *wt* CTLGA9 and Cry11Aa with APN. Three dimensional structures were modeled using Phyre-2 engine and molecular docking was performed by Discovery Studio 2.5. **(A)** Residues in APN that are involved in interaction with Cry11Aa (colored in green), with CTLGA9 (colored in red), or with both Cry11Aa and CTLGA9 (colored in yellow). **(B)** Residues (blue color) in CTLGA9 involved in binding with APN receptor. **(C)** Residues (blue color) in Cry11Aa involved in binding with APN.

**Figure 2:**
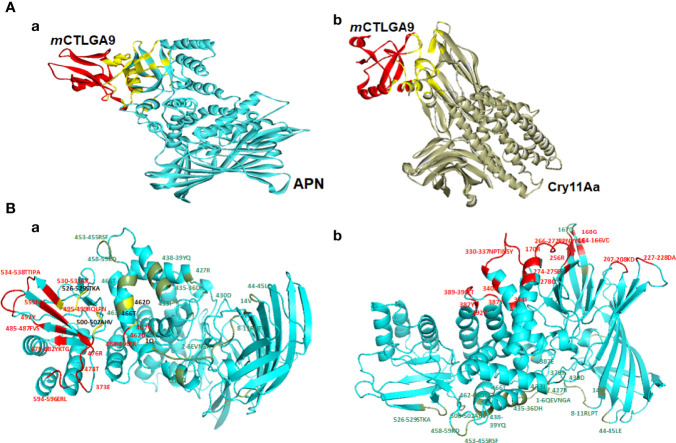
Molecular docking of mutant CTLGA9 with APN and Cry11Aa. Three-dimensional structures were modeled using Phyre-2 engine and molecular docking was performed by Discovery Studio 2.5. **(A-a)** Binding sites (yellow color) in docking complexes of m CTLGA9 with APN and **(A-b)** Cry11Aa. **(B)** Binding interface in APN receptor. **(B-a)** Residues in APN that are involved in interaction with *wt* CTLGA9 (colored in red) and Cry11Aa (colored in green) while yellow color showed common binding sites of both CTLGA9 and Cry11Aa in APN receptor interface **(B-b)** Residues in APN that are involved in interaction with *m* CTLGA9 (colored in red) and Cry11Aa (colored in green).

The docking complexes of *wt* CTLGA9 and Cry11Aa bound with the receptor APN and were analyzed using Discovery Studio, where it was found that the two ligand proteins had common APN binding sites (**Figure 1A** and **Table S1**). Further residues in *wt* CTLGA9 and Cry11Aa were analyzed involved in binding with APN (**Figures 1B, C**). The predicted hot spot residues involved in interaction between CTLGA9 and APN were L6, Q12, Y107, C109, and E110. L6 and Q12 were selected for alanine substitution mutation and the binding of the two resultant mutants with APN was checked through docking simulation analysis. Importantly, it was found that the mutation at Q12 was not associated with any change in Cry11Aa toxicity in the larvae bioassay. The effect was similar to the *wt* CTLGA9. By contrast, in the bioassay involving CTLGA9 mutated at L6, the toxicity of Cry11Aa was enhanced relative to that of the toxin mixed with *wt* CTLGA9. Therefore, CTLGA9 mutated at L6 was further characterized. Ramachandran analysis of *m* CTLGA9 predicted 95% of the residues to be in the favoured region according to phi and psi angle positions, whereas 5.4% of the residues were in the outlier region (**Supplementary Figure S1A**). The Z-score (-4.46) and energy value obtained from the ProSA-web server were below 0 and the interactions were energetically stable (**Supplementary Figures S1B, C**). The three-dimensional models of CTLGA9 (90% of sequences and 105 residues) were modeled with 99.9% confidence using the Phyre-2online server (**Supplementary Figure S1D**). Docking complexes of *m* CTLGA9 with APN receptor and Cry11Aa were obtained through GRAMM-X Protein-Protein Docking Web Server v.1.2.0 (http://vakser.compbio.ku.edu/resources/gramm/grammx/) and analyzed using Discovery Studio 2.5 software. The yellow colour showed the binding sites of *m* CTLGA9 to APN and Cry11Aa (**Figures 2A-a, b**), Notably, analysis of the docking complexes revealed that none of the residues in *m* CTLGA9 were in competition with those of Cry11Aa for binding to APN interface, unlike the residues of the *wt* CTLGA9 (**Figures 2B (a, b)** and **Table S2**).

### Expression and Purification of Mutant CTLGA9

**Figure 3:**
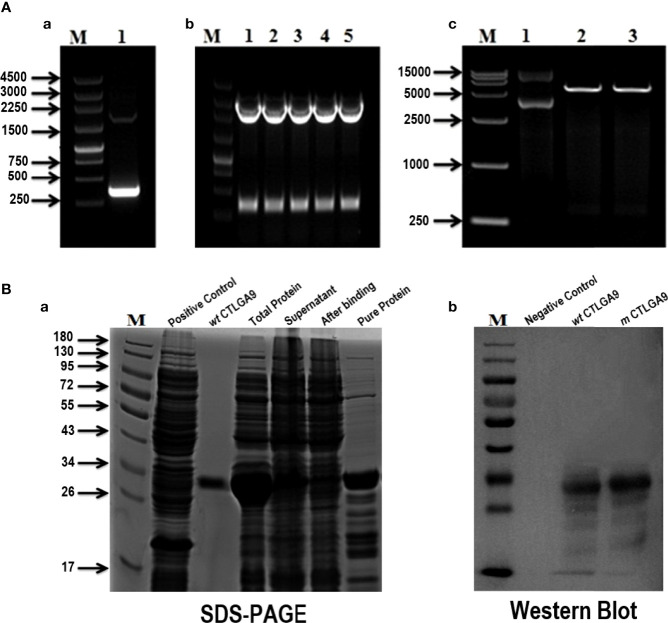
PCR Amplification of mutant CTLGA9 gene (A-a), Lane M: 250bp DNA marker (Thermo); Lane 1: amplified fragment of 354bp. **(A-b)** pMD18T recombinant plasmid digested with *NcoI/HindIII*, Lane M: 250bp DNA marker; Lane 1-5: plasmid digested product. **(A-c)** pET32α harboring *m* CTLGA9 recombinant plasmid digestion with enzyme sites *NcoI/HindIII*. Lane M: DL15000bp DNA marker (Thermo), Lane 1: Recombinant plasmid of CTLGA9- pET32α; Lane 2, 3: digested product. **(B)** SDS-PAGE and Western blotting analysis of *m* CTLGA9. **(B-a)** Lane M: 180 kDa molecular mass marker, Lane 2: Trx control; Lane 3: *wt* CTLGA9 protein; Lane 4: total mutant protein; Lane 5: mutant Supernatant; Lane 6: after binding sample; Lane 7: purified *m* CTLGA9 protein. **(B-b)** Western blot of *m* CTLGA9 protein. Lane M: 180 kDa molecular mass marker (Thermo); Lane 2: Trx control; Lane 3:*wt* CTLGA9 control protein; Lane 4: *m* CTLGA9 protein. Proteins were detected through CTLGA9 specific antibody.

Protein sequence analysis of *m* CTLGA9 indicated to be a 17 kDa stable protein, similar to the *wt* CTLGA9 protein. After amplification with gene-specific primers, the PCR product appeared as a band of approximately 354 bp (**Figures 3A-a**). The DNA fragment was extracted from the gel and ligated into the pMD18T cloning vector. **Figures 3A-b** shows confirmation that the plasmids had been correctly digested at the *NcoI/HindIII* enzyme sites. After insertion of the cloning vector into the pET32α vector, and transfer of the recombinant plasmid into *E.coli* BL21 competent cells. Subsequent gel analysis of the plasmids verified that the 354 bp fragment had been successfully inserted into pET32α (**Figures 3A-c**). Finally, SDS-PAGE separation of *m* CTLGA9 and *wt* CTLGA9 proteins that had been extracted using Ni-NTA chromatography confirmed them to be specific protein bands of approximately 33 kDa in size (**Figures 3B-a**). Further, confirmation through western blotting with specific polyclonal antibodies of *m* CTLGA9 revealed a band of 33 kDa on the PVDF membrane, which was consistent with the size of the *wt* CTLGA9 positive control (**Figures 3B-b**).

### Binding Activity of Mutant CTLGA9 With APN and Cry11Aa

**Figure 4:**
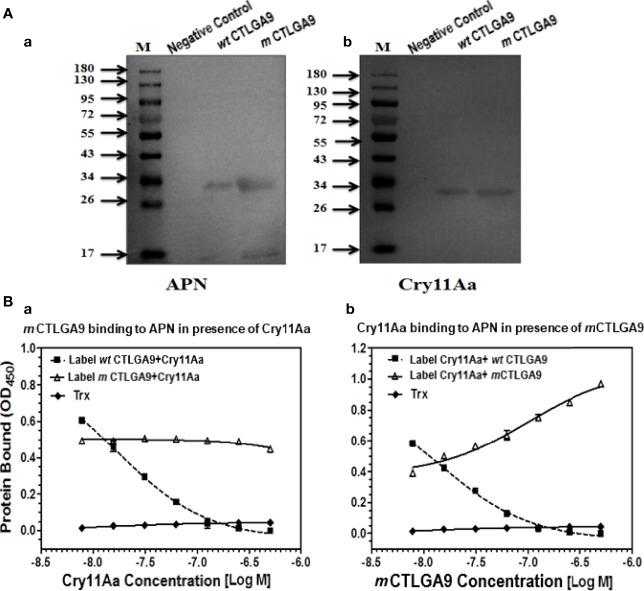
The binding activity of mutant CTLGA9 protein with APN receptor and Cry11Aa toxin protein. **(A (a, b))** Lane M: 180 kDa molecular mass marker (Thermo); Lane 2: Trx negative control; Lane 3: *wt* CTLGA9 (positive control); Lane 4: *m* CTLGA9. **(B-a)** Binding of biotinylated *m* and *wt* CTLGA9 (10 nm) to immobilized APN in the presence of increasing concentrations of unlabeled Cry11Aa (0-500nm); **(B-b)** Binding of biotinylated Cry11Aa to immobilized APN in the presence of increasing concentrations of unlabeled mutant and *wt* CTLGA9.

To determine the binding activity of *m* CTLGA9 with APN and Cry11Aa, ligand blotting and ELISA were performed. On the western blot membrane, *m* CTLGA9 that had been probed with either APN or Cry11Aa appeared a sharp band of approximately 33 kDa, similar to the positive control *wt* CTLGA9 (**Figures 4A (a, b)**), proving that the expressed *m* CTLGA9 could bind with APN and Cry11Aa proteins. The competitive ELISA based binding assay was performed to evaluate whether *m* CTLGA9 and Cry11Aa competed for binding to APN. The results clearly showed that labeled *m* CTLGA9 mixed with increasing concentrations of unlabeled Cry11Aa did not compete with Cry11Aa for the binding to fixed APN (**Figures 4B-a**). By contrast labeled *wt* CTLGA9 (as a positive control) mixed with same concentrations of unlabeled Cry11 Aa was found to compete with Cry11Aa for binding to APN (**Figures 4B-a**). On another side labeled Cry11Aa (10 nM) mixed with increasing concentration of *m* CTLGA9 (0–500 nM) was not competing to bind with fixed APN receptor proteins as compared to labeled Cry11Aa mixed with increasing concentration of *wt* CTLGA9 (0–500 nM) (**Figures 4B-b**). Non-significant binding activity was recorded between the control Trx and APN (**Figure 4B**).

### Toxicity Bioassay and Growth Curve of *m* CTLGA9

**Figure 5:**
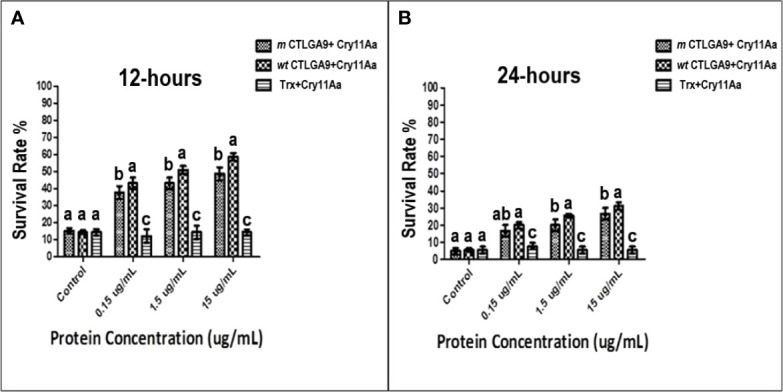
Feeding assay of Cry11Aa in the presence of recombinant *m* CTLGA9 fusion protein. *A. aegypti* larvae were fed with purified Cry11Aa (0.85 μg/mL) mixed with increasing concentrations of recombinant and control protein as *wt* CTLGA9 or Trx protein (0.15, 1.5, 15 μg/mL), and the mosquito larvae survival rate was noted at 12 h **(A)** and 24 h **(B)** of treatment with three replicates. Significance of difference was calculated by one way ANOVA followed by Tukey HSD test using IBM SPSS version 22 **(A, B)**, and identical letters are not significantly different (p > 0.05) while different letters indicate significant difference (p < 0.05).

To determine whether *m* CTLGA9 can alter the activity of Cry toxins, third instar larvae of *A. aegypti* were fed purified recombinant *m* CTLGA9 protein, *wt* CTLGA9 protein or Trx protein (as a control), and their cumulative survival rates were recorded. The bioassay results confirmed that *m* CTLGA9 could enhance Cry11Aa toxicity at 12 h (**Figure 5A**) and 24 h post-feeding (**Figure 5B**), compared with the effects of to *wt* CTLGA9 protein. In particular, at high concentration of *m* CTLGA9, significantly fewer larvae survived compared with those fed the same concentrations of *wt* CTLGA9 protein. Taken together, these results suggest that *m* CTLGA9 can enhance the larvicidal activity of Cry toxins.

The growth patterns of all three strains (recombinant *E. coli*) were more or less similar. The growth of bacteria carrying *m* CTLGA9 followed a steady curve, whereas the strain carrying *wt* CTLGA9 showed quicker growth up to 10–14 h albeit. The comparative rise for the later was approximately 10%, which can be considered a transient increase. Beyond 20 h, the growth patterns were similar for all strains; although the *wt* CTLGA9 carrying recombinant showed a slightly higher overall concentrations of cells (**Supplementary Figure S2**).

## Discussion

*B. thuringiensis* subspecies *israelensis* produces different Cry and Cyt family toxins used for mosquito control and management (27). Among these, Cry11Aa has proven to be one of the most active mosquitocidal agents against *A. aegypti* (28). Although the interactions between Cry toxins and their receptors have been relatively well studied, those between the midgut proteins and Cry toxins and toxin receptors remain unexplored. Molecular docking simulation is a highly useful technique for determining such protein-protein interactions (29, 30). Cry11Aa has been found to be bound to four different proteins in the brush border membrane vesicles of *A. aegypti* (31), two of which were identified as Cry11Aa functional receptors, namely the 65 kDa glycosylphosphatidyl-inositol-anchored protein ALP and the 73 kDa receptor protein APN (25, 32). Cry toxins initially bind to various receptor proteins on the host cell membrane and cause pore formation that leads to cell death. These toxins are made up of three domains. Among, which domain II is the most variable with regard to toxin specificity while binding toward the various receptors. Both the carbohydrate recognition domain of lectin and domain II of Cry toxins exhibit a β-prism structure (33–36), with exposed loop regions involved in receptor binding. Therefore on the basis of protein–protein interaction observations and predictions of the hot-spot residues in the receptor–ligand complex, a site-directed point mutation was introduced into the nucleotide sequence of *A. aegypti* CTLGA9 in this study, and the mutant protein was expressed to investigate its interaction with APN. C-type lectins belong to a large family of pattern recognition receptors that bind to galactose and mannose-type carbohydrates in the presence of calcium ions (37). Therefore, lectin proteins can recognize the structures of specific carbohydrates on cell surface and bind to them resulting in cell agglutination (38). Previously, it was reported that *A. aegypti* CTL-20 and galectin-14 could compete with Cry11Aa for binding to the receptor ALP1resulting in a significant increase in the the survival of mosquito larvae after being treated with Cry toxins (18, 26). In this study, the key amino acids involved in the binding of Cry11Aa or CTLGA9 to APN were determined, and the Leucine-6 residue was selected for alanine substitution mutation. Analysis of the docking complexes revealed that none of the residues in *m* CTLGA9 were involved in competition with Cry11Aa for binding to the APN interface. Our results showed that the expressed *m* CTLGA9 could bind equally with Cry11Aa and APN. ELISA binding assay revealed that labeled *m* CTLGA9 mixed with an increasing concentration of unlabeled Cry11Aa did not compete for binding toAPN. However, competitive binding to APN was detected in the positive control sample of labeled *wt* CTLGA9, mixed with variable concentrations of Cry11Aa. Furthermore, the bioassay results indicated that *m* CTLGA9 could enhance the toxicity of Cry proteins, as fewer *m* CTLGA9-fed larvae than *wt* CTLGA9-fed larvae survived at a high concentration of protein feeding. However, other CTLs have been found to bind not only to APN but also to other important receptors such as ALP and inhibiting Cry11Aa toxicity toward *A. aegypti* (18, 26). The toxicity assay also demonstrated that CTLGA9 protein was resistant to the insecticidal activity of Cry11Aa against *A. aegypti*. In conclusion, *wt* CTLGA9 can block Cry11Aa from binding to APN, which might decrease the mosquitocidal activity of the toxin as compared with the non-competitive effect of *m* CTLGA9. This mutated CTL protein may serve as a reference for future investigations of mechanism of *B. thuringiensis* Cry-based toxicity and the development of new biopesticides for mosquito control.

## Data Availability Statement

The original contributions presented in the study are included in the article/**Supplementary Material**. Further inquiries can be directed to the corresponding author.

## Author Contributions

The listed authors in the manuscript, LLZ, IA, and KB had the idea and designed the study. LLZ supervised the study. XG and LLZ provided technical support and vigorous guidance. KB and IA did the experiments and statistical analysis. IA and KB wrote the manuscript. IA, and LLZ revised and edit manuscript. All authors revised the manuscript and approved the final version before submission.

## Funding

This work was funded by the United Fujian Provincial Health and Education Project for Tackling Key Research (Grant No. 2019-WJ-29); Natural Science Foundation of Fujian Province (Grant No. 2020J01550 and 2020I0031); the Special Fund for Scientific and Technological Innovation of Fujian Agriculture and Forestry University (KFA20124A).

## Conflict of Interest

The authors declare that the research was conducted in the absence of any commercial or financial relationships that could be construed as a potential conflict of interest.

## Publisher’s Note

All claims expressed in this article are solely those of the authors and do not necessarily represent those of their affiliated organizations, or those of the publisher, the editors and the reviewers. Any product that may be evaluated in this article, or claim that may be made by its manufacturer, is not guaranteed or endorsed by the publisher.

## References

[1] Gabiane G Yen PS Failloux AB. Aedes Mosquitoes in the Emerging Threat of Urban Yellow Fever Transmission. Rev Med Virol (2022) 1:e2333. doi: 10.1002/rmv.2333 PMC9541788 35124859

[2] Santos S Smania-Marques R Albino VA Fernandes ID Mangueira FFA Altafim R. Prevention and Control of Mosquito-Borne Arboviral Diseases: Lessons Learned From a School-Based Intervention in Brazil (Zikamob). BMC Public Health (2022) 22:1–16. doi: 10.1186/s12889-022-12554-w 35135522 PMC8822808

[3] Zhao L Alto BW Duguma D. Transcriptional Profile for Detoxification Enzymes AeaGGT1 and AaeGGT2 From *Aedes Aegypti* (Diptera: Culicidae) in Response to Larvicides. J Med Entomol (2017) 54:878–87. doi: 10.1093/jme/tjw244 28399278

[4] Paiva MH Lovin DD Mori A Melo-Santos MA Severson DW Ayres CF. Identification of a Major Quantitative Trait Locus Determining Resistance to the Organophosphate Temephos in the Dengue Vector Mosquito. Aedes aegypti Genomics (2016) 107:40–8. doi: 10.1016/j.ygeno.2015.11.004 26576515

[5] Abbasi E Vahedi M Bagheri M Gholizadeh S Alipour H Moemenbellah-Fard MD. Monitoring of Synthetic Insecticides Resistance and Mechanisms Among Malaria Vector Mosquitoes in Iran: A Systematic Review. Heliyon (2022) 8:e08830. doi: 10.1016/j.heliyon.2022.e08830 35128113 PMC8808063

[6] Amelia-Yap ZH Azman AS Abubakar S Low VL. Streptomyces Derivatives as an Insecticide: Current Perspectives, Challenges and Future Research Needs for Mosquito Control. Acta Trop (2022) 229:1081. doi: 10.3389/fpls.2015.01081 35183537

[7] Vivekanandhan P Swathy K Murugan AC Krutmuang P. Insecticidal Efficacy of *Metarhizium Anisopliae* Derived Chemical Constituents Against Disease-Vector Mosquitoes. J Fungi (2022) 8:300. doi: 10.3390/jof8030300 PMC8950813 35330302

[8] Melo ALDA Soccol VT Soccol CR. *Bacillus Thuringiensis*: Mechanism of Action, Resistance, and New Applications: A Review. Crit Rev Biotechnol (2016) 36:317–26. doi: 10.3109/07388551.2014.960793 25264571

[9] Vega-Cabrera A Cancino-Rodezno A Porta H Pardo-Lopez L. Aedes Aegypti Mos20 Cells Internalizes Cry Toxins by Endocytosis, and Actin has a Role in the Defense Against Cry11Aa Toxin. Toxins (2014) 6:464–87. doi: 10.3390/toxins6020464 PMC3942746 24476709

[10] Lee S-B Chen J Aimanova KG Gill SS. Aedes Cadherin Mediates the *In Vivo* Toxicity of the Cry11Aa Toxin to Aedes Aegypti. Peptides (2015) 68:140–7. doi: 10.1016/j.peptides.2014.07.015 PMC4305047 25064814

[11] Pardo-Lopez L Soberon M Bravo A. *Bacillus Thuringiensis* Insecticidal Three-Domain Cry Toxins: Mode of Action, Insect Resistance and Consequences for Crop Protection. FEMS Microbiol Rev (2013) 37:3–22. doi: 10.1111/j.1574-6976.2012.00341.x 22540421

[12] Li J Carroll J Ellar DJ. Crystal Structure of Insecticidal δ-Endotoxin From *Bacillus Thuringiensis* at 2.5 Å Resolution. Nature (1991) 353:815–21. doi: 10.1038/353815a0 1658659

[13] Morse RJ Yamamoto T Stroud R. Structure of Cry2Aa Suggests an Unexpected Receptor Binding Epitope. Structure (2001) 9:409–17. doi: 10.1016/S0969-2126(01)00601-3 11377201

[14] Guo S Ye S Liu Y Wei L Xue J Wu H. Crystal Structure of *Bacillus Thuringiensis* Cry8Ea1: An Insecticidal Toxin Toxic to Underground Pests, the Larvae of *Holotrichia Parallela* . J Struct Biol (2009) 168:259–66. doi: 10.1016/j.jsb.2009.07.004 19591941

[15] Hui F Scheib U Hu Y Sommer RJ Aroian RV Ghosh P. Structure and Glycolipid Binding Properties of the Nematicidal Protein Cry5B. Biochemistry (2012) 51:9911–21. doi: 10.1021/bi301386q PMC3567309 23150986

[16] Bravo A Gill SS Soberon M. Mode of Action of *Bacillus Thuringiensis* Cry and Cyt Toxins and Their Potential for Insect Control. Toxicon (2007) 49:423–35. doi: 10.1016/j.toxicon.2006.11.022 PMC1857359 17198720

[17] Palma L Muñoz D Berry C Murillo J Caballero P. *Bacillus Thuringiensis* Toxins: An Overview of Their Biocidal Activity. Toxins (2014) 6:3296–325. doi: 10.3390/toxins6123296 PMC4280536 25514092

[18] Zhang L-L Hu X-H Wu S-Q Batool K Chowdhury M Lin Y. *Aedes Aegypti* Galectin Competes With Cry11Aa for Binding to ALP1 To Modulate Cry Toxicity. JAFC (2018) 66:13435–43. doi: 10.1021/acs.jafc.8b04665 30556692

[19] Batool K Alam I Jin L Xu J Wu C Wang J. CTLGA9 Interacts With ALP1 and APN Receptors To Modulate Cry11Aa Toxicity in *Aedes Aegypti* . JAFC (2019) 67:8896–904. doi: 10.1021/acs.jafc.9b01840 31339308

[20] Mcconnell MT Lisgarten DR Byrne LJ Harvey SC Bertolo E. Winter Aconite (*Eranthis Hyemalis*) Lectin as a Cytotoxic Effector in the Lifecycle of *Caenorhabditis Elegans* . Peer J (2015) 3:e1206. doi: 10.7717/peerj.1206 26312191 PMC4548470

[21] Hu X Zhang X Zhong J Liu Y Zhang C Xie Y. Expression of Cry1Ac Toxin-Binding Region in *Plutella Xyllostella* Cadherin-Like Receptor and Studying Their Interaction Mode by Molecular Docking and Site-Directed Mutagenesis. Int J Biol Macromol (2018) 111:822–31. doi: 10.1016/j.ijbiomac.2017.12.135 29305214

[22] Berdougo E Doranz BJ. High-Throughput Alanine Scanning: Epitope Mapping and Engineering Complex Membrane Proteins by Comprehensive Mutagenesis. J Genet Eng Biotechnol (2012) 32:30–1. doi: 10.1089/gen.32.12.12

[23] Bradford MM. A Rapid and Sensitive Method for the Quantitation of Microgram Quantities of Protein Utilizing the Principle of Protein-Dye Binding. Anal Biochem (1976) 72:248–54. doi: 10.1016/0003-2697(76)90527-3 942051

[24] Zhang L Zhao G Hu X Liu J Li M Batool K. Cry11Aa Interacts With the ATP-Binding Protein From *Culex Quinquefasciatus* to Improve the Toxicity. JAFC (2017) 65:10884–90. doi: 10.1021/acs.jafc.7b04427 29215274

[25] Chen J Likitvivatanavong S Aimanova KG Gill SS. A 104 kDa *Aedes Aegypti* Aminopeptidase N is a Putative Receptor for the Cry11Aa Toxin From *Bacillus Thuringiensis* Subsp. Israelensis. Insect Biochem Mol Biol (2013) 43:1201–8. doi: 10.1016/j.ibmb.2013.09.007 PMC3872109 24128608

[26] Batool K Alam I Zhao G Wang J Xu J Yu X. C-Type Lectin-20 Interacts With ALP1 Receptor to Reduce Cry Toxicity in *Aedes Aegypti* . Toxins (2018) 10:390. doi: 10.3390/toxins10100390 PMC6215184 30257487

[27] Ben-Dov E. *Bacillus Thuringiensis* Subsp. Israelensis and its Dipteran-Specific Toxins. Toxins (2014) 6:1222–43. doi: 10.3390/toxins6041222 PMC4014730 24686769

[28] Chilcott CN Ellar DJ. Comparative Toxicity of *Bacillus Thuringiensis* Var. Israelensis Crystal Proteins *In Vivo* and *In Vitro* . Microbiology (1988) 134:2551–8. doi: 10.1099/00221287-134-9-2551 3254944

[29] Ahmad A Javed MR Rao AQ Khan MA Ahad A Shahid AA. In-Silico Determination of Insecticidal Potential of Vip3Aa-Cry1Ac Fusion Protein Against Lepidopteran Targets Using Molecular Docking. Front Plant Sci (2015) 6:1081. doi: 10.3389/fpls.2015.01081 26697037 PMC4667078

[30] Rosenfeld L Heyne M Shifman JM Papo N. Protein Engineering by Combined Computational and *In Vitro* Evolution Approaches. Trends Biochem Sci (2016) 41:421–33. doi: 10.1016/j.tibs.2016.03.002 27061494

[31] Fernandez LE Martinez-Anaya C Lira E Chen J Evans A Hernández-Martínez S. Cloning and Epitope Mapping of Cry11Aa-Binding Sites in the Cry11Aa-Receptor Alkaline Phosphatase From *Aedes Aegypti* . Biochemistry (2009) 48:8899–907. doi: 10.1021/bi900979b PMC3704566 19697959

[32] Chen J Aimanova KG Pan S Gill SS. Identification and Characterization of *Aedes Aegypti* Aminopeptidase N as a Putative Receptor of *Bacillus Thuringiensis* Cry11A Toxin. Insect Biochem Mol Biol (2009) 39:688–96. doi: 10.1016/j.ibmb.2009.08.003 PMC2763025 19698787

[33] Bourne Y Roig-Zamboni V Barre A Peumans WJ Astoul CH Van Damme EJ. The Crystal Structure of the *Calystegia Sepium* Agglutinin Reveals a Novel Quaternary Arrangement of Lectin Subunits With a β-Prism Fold. J Biol Chem (2004) 279:527–33. doi: 10.1074/jbc.M308218200 14561768

[34] Fernández LE Pérez C Segovia L Rodríguez MH Gill SS Bravo A. Cry11Aa Toxin From *Bacillus Thuringiensis* Binds its Receptor in *Aedes Aegypti* Mosquito Larvae Through Loop α-8 of Domain II. FEBS Lett (2005) 579:3508–14. doi: 10.1016/j.febslet.2005.05.032 15963509

[35] Meagher JL Winter HC Ezell P Goldstein IJ Stuckey JA. Crystal Structure of Banana Lectin Reveals a Novel Second Sugar Binding Site. Glycobiology (2005) 15:1033–42. doi: 10.1093/glycob/cwi088 15944373

[36] Fernandez LE Aimanova KG Gill SS Bravo A Soberón M. A GPI-Anchored Alkaline Phosphatase is a Functional Midgut Receptor of Cry11Aa Toxin in *Aedes Aegypti* Larvae. Biochem J (2006) 394:77–84. doi: 10.1042/BJ20051517 16255715 PMC1386005

[37] Drickamer K. Engineering Galactose-Binding Activity Into a C-Type Mannose-Binding Protein. Nature (1992) 360:183. doi: 10.1038/360183a0 1279438

[38] Ayaad TH Al-Akeel RK Olayan E. Isolation and Characterization of Midgut Lectin From *Aedes Aegypti* (L.) (Diptera: Culicidae). Braz Arch Biol Technol (2015) 58:905–12. doi: 10.1590/S1516-89132015060277

