# Supplementary material for: Function of CTLGA9 Amino Acid Residue Leucine-6 in Modulating Cry Toxicity
Source: Front Immunol. 2022 Jul 5;13:906259. doi: 10.3389/fimmu.2022.906259 (PMC9294448; doi:10.3389/fimmu.2022.906259)
Supplement: Supplementary file 1 [file DataSheet_1.docx]

Supplementary Material

# **Supplementary** Figures

**
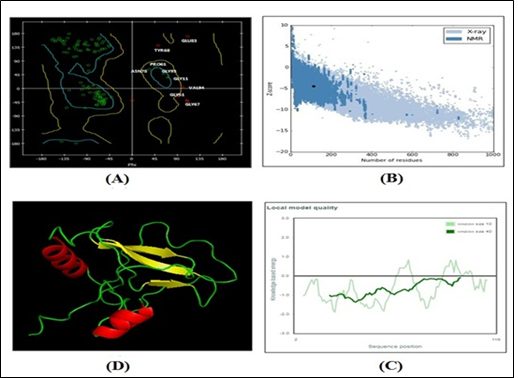
**

**Supplementary Figure 1.** (A) Ramachandran plot analysis generated using the software Discovery studio 2.5 Server. (B) Energy curve (C) Z–score analysis (D) Predicted 3D structure of mutant CTLGA9 by Phyre-2 Server.


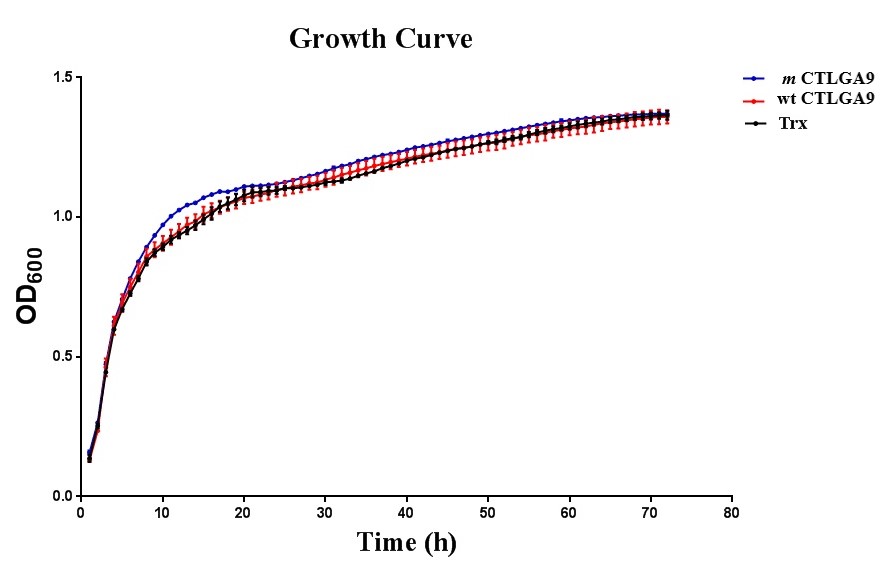


**Supplementary Figure 2.** Growth curve of *m* CTLGA9 (blue color), *wt* CTLGA9 (red color) and control Trx (blake color) was measured for 0-72 h at 37°C.

# **Supplementary** Tables

**Table S1** Residues in APN receptor that is involved in binding to CTLGA9 and Cry11Aa.

| **Ligand Protein** | **APN residues involve in binding** | **Common APN sites binding to CTLGA9 and Cry11Aa** |
| --- | --- | --- |
| ***wt* CTLGA9** | 1Q, 373E, 462D, 466-67TV, 476R, 479-82YKTG, 485-87FVS, 489-90ER, 492Y, 495-502RQLPNAHV, 526-31STKAAK, 534-38TTIPA, 555N, 594-96ERL | 1Q, 462D, 500-502AHV, 526-529STKA |
| **Cry11Aa** | 1-6QEVNGA, 8-11RLPT, 14V, 44-45LE, 56-57RL, 325D, 329Y, 379T, 387E, 427R, 430D, 433I, 435-36DH, 438-39YQ, 453-55RSF, 458-459KD, 463-64DS, 466T, 500-502AHV, 504H, 526-529STKA |  |

**Table S2** Overlapping binding sites of APN with *wt* and *m* CTLGA9 along with Cry11Aa.

| **Ligand protein** | **Binding sites of APN with ligand protein** | **Binding sites of APN with Cry11Aa** | **Overlapping binding sites of APN interface** |
| --- | --- | --- | --- |
| ***wt* CTLGA9** | 1Q, 373E, 462D, 466-67TV, 476R, 479-82YKTG, 485-87FVS, 489-90ER, 492Y, 495-502RQLPNAHV, 526-31STKAAK, 534-38TTIPA, 555N, 594-96ERL | 1-6QEVNGA, 8-11RLPT, 14V, 44-45LE, 56-57RL, 325D, 329Y, 379T, 387E, 427R, 430D, 433I, 435-36DH, 438-39YQ, 453-55RSF, 458-459KD, 463-64DS, 466T, 500-502AHV, 504H, 526-529STKA | 1Q, 462D, 500-502AHV, 526-529STKA |
| ***m* CTLGA9** | 164-166VDD, 158G, 170R, 207-208KD, 227-228DA, 256R, 266-72RPNVYGE, 274-75EF, 278Q, 330-337FNPTINSY, 344I, 389-390ET, 392Y |  | Nil |
